# Synthesis of Ni/NiAlO_x_ Catalysts for Hydrogenation Saturation of Phenanthrene

**DOI:** 10.3389/fchem.2021.757908

**Published:** 2021-10-08

**Authors:** Dao-Cheng Liu, Yu Chen, Jie-Ying Jing, Antony Rajendran, Hong-Cun Bai, Wen-Ying Li

**Affiliations:** ^1^ State Key Laboratory of Clean and Efficient Coal Utilization, Taiyuan University of Technology, Taiyuan, China; ^2^ Key Laboratory of Coal Science and Technology Ministry of Education, Taiyuan University of Technology, Taiyuan, China; ^3^ State Key Laboratory of High-efficiency Utilization of Coal and Green Chemical Engineering, Ningxia University, Yinchuan, China

**Keywords:** phenanthrene hydrogenation, metallic electron-deficient state, nickel aluminate, calcination temperature, steric hindrance, competitive adsorption

## Abstract

The saturation of octahydrophenanthrene was the rate-determining step in the hydrogenation process from phenanthrene to perhydrophenanthrene, which was due to the steric hindrance and competitive adsorption of octahydrophenanthrene. In this work, a series of Ni/NiAlO_x_ catalysts with a uniform electron-deficient state of Ni derived from the nickel aluminate structure was synthesized to overcome the disadvantage of noble catalyst and the traditional sulfided catalysts in the saturation hydrogenation process of phenanthrene. Results showed that the catalyst calcinated at 650°C possessed more Ni^2+^ (∼98%) occupying octahedral sites and exhibited the highest r_obs_ (1.53 × 10^−3^ mol kg^−1^ s^−1^) and TOF (14.64 × 10^−3^ s^−1^) for phenanthrene hydrogenation. Furthermore, its ability to overcome steric hindrance and promote the rate-determining step was proven by octahydrophenanthrene hydrogenation. Comparing the evolution of hydrogenation activity with the change in the electronic structure of surface Ni sites, it was shown that the increase of metallic electron deficiency hindered the π-back bonding between surface Ni and aromatic rings, which was unfavorable for aromatic adsorption. As a result, the phenanthrene hydrogenation saturation performance can be enhanced by stabilizing the electron-deficient state of surface Ni on an optimal degree.

## Introduction

Polycyclic aromatic hydrocarbons (PAHs) are predominantly present in coal tar, whose direct discharge and insufficient combustion would cause environmental pollution (Hayakawa et al., 2000; Li and Suzuki 2010). Therefore, the treatment of PAHs has gained undivided attention, and hydrogenation of PAHs is an attractive method as it effectively yields the cycloalkanes which can be blended into the jet fuels to improve the volumetric calorific value and combustion performance (Yoon et al., 1996; Zhang et al., 2018; Jing et al., 2020; Jia et al., 2021). However, it is quite difficult to achieve complete saturation while performing the hydrogenation of PAHs with more aromatic rings, and in particular, the saturation of the final aromatic ring is more difficult (Beltramone et al., 2008). To solve this problem, a variety of catalysts have been attempted to promote the hydrogenation of PAHs using phenanthrene (PHE) as a typical PAH compound. PHE has three aromatic rings, and its hydrogenation process is typically exothermic and consecutive. During the hydrogenation of PHE, the complete saturation, that is, the higher selectivity of perhydrophenanthrene (PHP), is rarely reported, with the exception of very costly noble bimetallic catalyst requiring the higher metal loadings (2.0 wt% Pt and 5.4 wt% Pd) (Qian et al., 1999). Considering the traditional sulfide catalysts (Ni(Co)Mo(W)S), the complete saturation of PHE is still a big challenge despite a list of strategies having been attempted (Fu et al., 2015; Jiang et al., 2017; Luo et al., 2018; Wang D et al., 2019). The complete saturation of PHE is determined by the hydrogenation of symmetric-octahydrophenanthrene (s-OHP) to PHP (the rate-determining step), whose rate is limited by the natural steric hindrance and unfavorable competitive adsorption of s-OHP between partially hydrogenated aromatics (Korre et al., 1995; Beltramone et al., 2008). In this milieu, the catalysts which are low cost without compromising the higher hydrogenation efficiency, especially for the conversion of s-OHP to PHP, have received much attention in the literature, but such kind of catalysts are still scarce.

Being the familiar low-cost hydrogenation catalysts with an excellent H_2_ dissociation capacity, Ni-based catalysts always seem to be the alternative choice for the group-VIII noble metal catalysts (Pd and Pt) (Hammer and Norskov 1995; De et al., 2016). However, though it accomplishes the excellent conversion of PHE, the Ni-based catalysts still struggle to attain the higher selectivity of PHP during the hydrogenation. For instance, the typical Ni/Al_2_O_3_ catalyst displays 97.9% conversion of PHE but achieves a very low selectivity of PHP (20.8%) (Nuzzi and Marcandalli 2003). This is because the adsorption energy of polycyclic aromatic hydrocarbons is higher over Ni (111) surface than that over Pt (111) (Yazdi et al., 2017). In line with the general adsorption mechanism of aromatics over the metallic surfaces, the adsorption of PHE over the Ni surface can be improved through σ bonding (electronic transfer from filled π molecular orbital of aromatics to atomic vacant d orbital of metallic Ni) and through a π-back bonding (electronic back-donation from filled atomic d orbital of Ni to vacant π* molecular orbital of aromatics) (Stanislaus and Cooper 1994). This underlines the importance of electron deficiency in metallic Ni active sites to establish the improved adsorption of PHE through σ bonds, which in turn directs to look for the method to tune the electronic properties of Ni active sites.

The electron transfer driven by differences in the Fermi level of the metal nanoparticles and the support is an important phenomenon in metal-support interaction (MSI). Therefore, enhancing the MSI would favorably influence the electronic properties of metallic active sites (van Deelen et al., 2019). NiAl_2_O_4_ spinel-based catalysts seem to be attractive because they offer the enhanced MSI between reduced Ni and oxide support due to the confinement effect of spinel precursors. As a result, the improved metallic Ni dispersion (Srifa et al., 2018), decreased practical size Rubén et al. (2012), and good resistance to coke formation (Salhi et al., 2011; Suffredini et al., 2017) can be envisaged. Deviating from the typical spinel structure, Ni^2+^ can occupy both the tetrahedral (Ni_tetra_) and octahedral cation sites (Ni_octa_) in NiAl_2_O_4_, that is, an inverse spinel structure. Furthermore, Ni_octa_ sites are essential as they exhibit strong interaction and better reducibility (Yancheshmeh et al., 2020).

Based on the above facts, in this work, a series of nickel aluminate–based catalysts with more Ni_octa_ sites were synthesized by the modified sol–gel method using citric acid. The facile method that varies calcination temperatures was selected to adjust Ni^2+^ distribution and the electronic state of Ni active sites based on the enhanced MSI. The catalytic activities of the prepared catalysts were evaluated in the hydrogenation of PHE as well as s-OHP, which provided the insights into the potential of present catalysts to overcome the steric hindrance associated with the hydrogenation of PAHs. Moreover, the role of electron deficiency in Ni active sites was emphasized and discussed.

## Experiment

### Preparation of Catalysts

Nickel aluminate–based catalysts were prepared by the modified sol–gel method using citric acid. Ni(NO_3_)_2_·6H_2_O and Al(NO_3_)_3_·9H_2_O were taken in a certain molar ratio of Ni:Al (1.45:2). The molar ratio of citric acid and water to metal ions (Ni^2+^ and Al^3+^) was 1:1 and 40:1, respectively. Ni(NO_3_)_2_·6H_2_O, Al(NO_3_)_3_·9H_2_O, and citric acid were initially dissolved in deionized water and stirred at room temperature for 3 h. Stirring was further continued but at 80°C for 6 h to ensure the condensation reaction. The resulting solid was kept overnight at room temperature and sequentially dried at two different conditions (100°C for 12 h and 120 °C for 12 h, yielding a green fluffy xerogel). The obtained solids were calcinated at different temperatures (550, 600, 650, 700, and 750°C) for 2 h in air and subsequently reduced at 520 °C for 5 h under H_2_ flow to produce the five different Ni/NiAlO_x_-T catalysts (T: calcination temperature). Pure NiO and Al_2_O_3_ were also prepared by exactly following the abovementioned sol–gel method and calcination procedure (650°C for 2 h), and mechanically mixed together with the same Ni/Al molar ratio to prepare the catalyst for comparison (NiO + Al_2_O_3_-mixed).

### Characterization

The powder X-ray diffraction (XRD) patterns of samples were recorded on a Rigaku Ultima IV multifunctional X-ray diffractometer (Rigaku, Japan) in the range of 10–90° at a rate of 4°min^−1^. For this, the monochromatic Cu Kα radiation was generated at 40 kV and 40 mA. The Scherrer formula was applied on the powder XRD data to calculate the crystallite size. Varian Cary 300 was used to record the diffuse reflectance UV-Vis spectra of powder samples in the range of 300–800 nm. The H_2_ temperature-programmed reduction (TPR) experiment was performed on Micromeritics AutoChem II 2920 analyzer. 0.1 g of the sample was pretreated at Ar atmosphere (300°C at 2 h) and then heated to 900°C at 10 °C min^−1^ in the presence of 10 vol% H_2_/Ar at a flow rate of 25 ml min^−1^. The transmission electron microscope (TEM) images of the samples were captured using FEI Tecnai G2 F30 to explore the morphologies of the as-synthesized catalysts at an accelerate voltage of 200 kV. For the calculation of particle size distribution, at least 300 different points of Ni particles were considered. The textural properties of the as-synthesized catalysts were analyzed using N_2_ adsorption–desorption measurements (Quantachrome Autosorb-iQ instrument) in a liquid N_2_ bath. The specific surface area (S_BET_) was estimated by Brunauer–Emmett–Teller (BET) equation. The microporous and mesoporous surface area values (S_mic_ and S_meso_) were estimated by t-plot. Fourier transform infrared spectra of the samples using CO as a probe molecule (CO-FTIR) were acquired on a Bruker TENSOR 27 spectrophotometer (4,000–400 cm^−1^, resolution 2 cm^−1^) equipped with an *in situ* stainless steel reactor. 10 mg catalyst was pressed into a wafer (Φ = 12.6 mm) and reduced *in situ* at 520°C for 5 h in H_2_ flow (50 ml min^−1^). The sample was cooled to 40°C under Ar, and then the gas flow was changed to 5 vol% CO/Ar that was maintained for 1 h to ensure the maximum CO adsorption. Prior to the analysis, the physically absorbed CO was purged under Ar flow (40 ml min^−1^). X-ray photoelectron spectra (XPS) of the samples were carried out on a Kratos analytical AXIS SUPRA equipped with an Al Kα (X-ray source). The CO pulse chemisorption was performed on Micromeritics AutoChem II 2920 instrument. 15 mg of the sample was put in a quartz tube and reduced *in situ* at 520°C for 5 h in H_2_ flow (50 ml min^−1^). After stabilizing the temperature of the reactor at 40°C under Ar flow, 5 vol % CO/Ar was pulsed into the reactor and repeated for 40 times with the sampling loop capacity of 0.1 ml. The element content of the as-synthesized samples was detected by inductively coupled plasma optical emission spectrometer (ICP‒OES, Agilent 5110).

### Catalytic Activity

The hydrogenation of PHE was carried out in a fixed-bed continuous-flow stainless steel reactor with 10 mm internal diameter and 500 mm length. The dried catalyst (60–80 mush) was diluted with silica sand to 2 mL before being loaded into the reactor. Both the ends of the catalyst bed were filled with additional silica sand of 2 mL. The catalyst was directly reduced in the fixed-bed reactor by heating to 520°C at a rate of 3°Cmin^−1^ in a H_2_ flow (50 ml min^−1^) and kept for 5 h. Then, the catalyst was naturally cooled to reaction temperature in a continuous H_2_ flow. The operating conditions for hydrogenation of PHE were as follows: total pressure (5.0 MPa), temperature (280°C), feed rate of 1.0 wt% PHE in decalin (6 ml h^−1^), flow rate of H_2_ (60 ml min^−1^), and the weight hourly space velocity (WHSV) of 52 h^−1^. The effect of external and internal diffusion was checked by adjusting the mass flow rate and particle diameter, respectively. The product was collected and analyzed using Shimadzu GC-2010 installed with RTX-5 column and flame ionization detector (FID). The products were further qualitatively analyzed with GC equipped with a mass spectrometer (Agilent GC-MS).

To investigate the effect of steric hindrance on hydrogenation of PHE, s-OHP was selected as the model compound instead of PHE, and the abovementioned experimental procedure was exactly followed. For the investigation of intrinsic activity of catalysts, the conversion of PHE and s-OHP was kept low by changing the values of WHSV.

Herein, the intrinsic hydrogenation activity of catalysts was observed in terms of the reaction rate (r_obs_, mol s^−1^kg^−1^) using Eq. 1.

$${\text{r}}_{obs}=\frac{dx}{d\left(W/F\right)},$$ (1)

where F is the reactant molar flow (mol s^−1^), x is the PHE conversion (%) or the conversion of s-OHP into PHP (%), and W is the catalyst mass (kg).

The hydrogenation turnover frequency (TOF, s^−1^) values of PHE and s-OHP were calculated from Eq. 2.

$$\mathrm{TOF}=\frac{F\times x}{N_{Ni}\times f_{Ni}},$$ (2)

where F is the reactant molar flow (mol s^−1^), x is the PHE conversion (%) or the conversion of s-OHP into PHP (%), N_Ni_ is the number of Ni atoms in the catalyst (mol), and *f*_*Ni*_ is the dispersion of Ni detected by CO pulse adsorption (%).

## Results and Discussion

### Properties of the As-Synthesized Catalysts

#### Element Composition and Textural Properties

The actual concentration of nickel and aluminum in the prepared spinel-based catalysts was determined by ICP-OES, and the actual Ni/Al ratio was accordingly calculated (Table 1). All the prepared catalysts displayed the same Ni/Al molar ratio which is equal to the theoretical Ni/Al ratio. This indicated that no loss of Al or Ni ions occurred during the preparation process regardless of calcination temperature. This also led to the assumption that the different physiochemical properties of prepared nickel aluminate samples might be attributed to the variation in the distribution of Ni_octa_ and Ni_tetra_ with the variation of calcination temperature. The Ni/Al molar ratio (0.73) of the prepared nickel aluminate samples was higher than that of typical nickel aluminate (NiAl_2_O_4_, Ni/Al molar ratio was 0.5), indicating that there were additional Ni^2+^ ions in the present spinel structures. This could be due to the inversion structure of the nickel aluminate spinel structure, in which the additional Ni^2+^ ions were occupied in the octahedral sites to provide more desirable active sites upon the reduction.

**TABLE 1:** Ni/Al molar ratio and surface properties of Ni/NiAlO_X_ catalysts with different calcination temperatures.

Catalyst	Ni/Al molar ratio^a^	After calcination			After reduction		
		${\text{S}}_{\mathrm{BET}}^{b}$ (m^2^ g^−1^)	${\text{S}}_{meso}^{c}$ (m^2^ g^−1^)	${\text{S}}_{mic}^{c}$ (m^2^ g^−1^)	${\text{S}}_{\mathrm{BET}}^{b}$ (m_2_ g^−1^)	${\text{S}}_{meso}^{c}$ (m^2^ g^−1^)	${\text{S}}_{mic}^{c}$ (m^2^ g^−1^)
Ni/NiAlO_X_-550	0.74	185.86	85.52	100.35	115.96	102.31	13.66
Ni/NiAlO_X_-600	0.74	181.75	102.10	79.65	129.55	129.55	—
Ni/NiAlO_X_-650	0.73	179.29	155.96	23.31	177.95	177.95	—
Ni/NiAlO_X_-700	0.73	169.44	155.08	14.36	165.33	165.33	—
Ni/NiAlO_X_-750	0.73	156.02	156.02	—	129.28	129.28	—

a Detected by ICP-OES. The theoretical Ni/Al molar ratio is 0.73.

b BET surface area.

c Calculated by t-plot. All of the samples are tableted at 15 MPa.

N_2_ physisorption was carried out to determine the S_BET_, S_micro_, and S_meso_ of synthesized catalysts. All the analyzed samples displayed the hysteresis loops corresponding to the mesoporous structure with no significant deviation in the pore diameter values. While increasing the calcination temperature of samples, the S_BET_ value was decreased from 185.86 to 156.02 m^2^/g, whereas the S_meso_ value was increased with a loss of S_micro_ and became stagnant after a certain calcination temperature (650^°^C) (Table 1). Compared to the reported nickel aluminate, the obtained higher surface area and porosity of the samples could be attributed to the evolution of CO_2_ from citric acid (Rogers et al., 2016; Miryam et al., 2017; Jing et al., 2018). Considering the textural properties of reduced catalysts, Ni/NiAlO_X_-650 exhibited the highest mesoporous surface area (177.95 m^2^/ g) by which Ni/NiAlO_X_-650 might possess a higher performance in the hydrogenation of PHE with a better mass transfer.

#### Analysis of Ni Occupation

**FIGURE 1:**
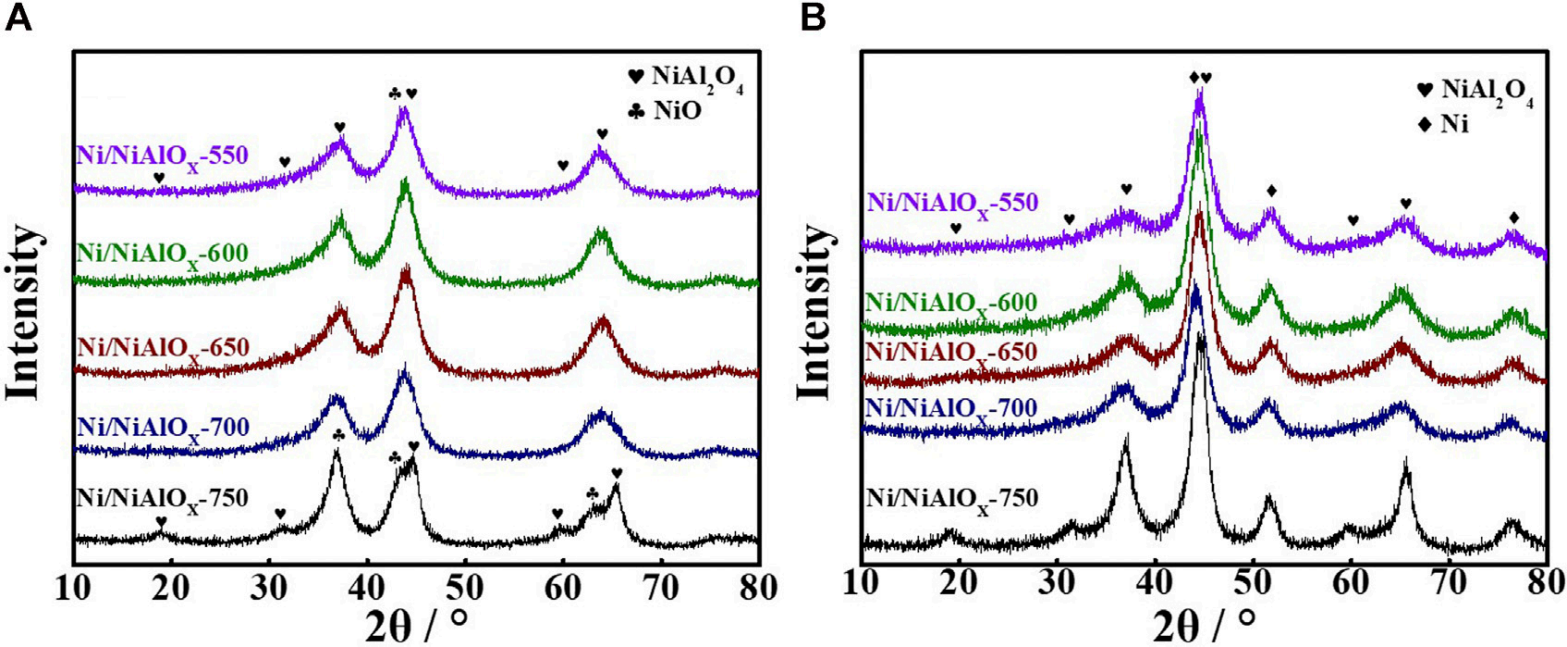
Powder XRD spectra of Ni/NiAlO_x_ catalysts after calcination **(A)** and reduction **(B)** at different temperatures.

In order to confirm the Ni occupation in the as-prepared catalyst, we conducted XRD characterization. Figure 1 displayed the powder XRD patterns of the samples before and after reduction. Compared with the standard peaks of NiO (PDF#78-0643) and NiAl_2_O_4_ (PDF#78-1601), the peaks of the samples calcined at the lower temperature (<750^o^C) were shifted (Figure 1a), which was related to the expansion in the nickel aluminate spinel structure. The expansion was derived from the replacement of smaller Al^3+^ (0.54 Å) by larger Ni^2+^ (0.69 Å) ions, especially at the octahedral sites. This phenomenon indicated that major Ni^2+^ ions occupied at the octahedral sites in the spinel structure. The occupancy of Ni^2+^ ions predominantly at the octahedral sites in the spinel structure can be evidenced by the larger intensity of the peak (45.0°) corresponding to the presence of Ni_octa_ sites as well as no intensity of the peak (31.4°) corresponding to the presence of Ni_tetra_ sites (Han et al., 2004; Tirsoaga et al., 2011; Miryam et al., 2017). On the other hand, for the sample prepared by calcining at 750^o^C, the appearance of characteristic powder XRD peaks of NiAl_2_O_4_ and NiO species is apparent. Especially, the diffraction peak at 31.4°, which belonged to Ni_tetra_, was obvious. It indicated that the higher calcination temperature promoted the occupancy of tetrahedral sites by Ni^2+^ ions (O'Neill et al., 1991). Meanwhile, the separation of NiO and Ni_tetra_ structures occurred at the higher calcination temperature. DR UV-Vis spectra of the calcined samples (Supplementary Figure S1) further proved this conclusion. The absorption bands of the stoichiometric NiAl_2_O_4_ spinel structure at 642 and 597 nm were not seen for the samples calcined less than 700^o^C. The intensity of these peaks was weak for the sample calcined at 700^o^C and became relatively strong in the case of the sample calcined at 750^o^C. This was in accordance with the XRD results and further indicated the formation of the stoichiometric NiAl_2_O_4_ spinel structure with Ni^2+^ occupying Ni_tetra_ sites in the samples calcined at temperature (≥700°C) according to the ion-exchange reaction $Ni_{\mathrm{octa}}+Al_{tetra}⇌Ni_{\mathrm{tetra}}+Al_{\mathrm{octa}}$ (Elias et al., 2019).

After the reduction of calcined samples at 520^o^C, the reduced samples were characterized by the powder XRD analysis (Figure 1b). The characteristic peak of metallic Ni species (PDF#65-2865) appeared with the obvious decrease in the intensity of the characteristic peak corresponding to Ni_octa_ sites. However, though the obvious decrease in the peak intensity was noticed, there were still the characteristic peaks of the nickel aluminate structure seen in Figure 1b. This suggested that those unreduced Ni^2+^ ions were present in the bulk nickel aluminate phase and served as the support. The peak corresponding to Ni(200) surface was considered for measuring the crystallite size (3 nm) of metallic Ni species by the Scherrer formula. This small crystallite size can be assigned to the strong MSI experienced by the predominant Ni_octa_ sites in the calcined samples, and these Ni^2+^ ions are supposedly reduced to small Ni^0^ sites with a higher dispersion.

#### Quantification Analysis of Different Ni^2+^ Species

**FIGURE 2:**
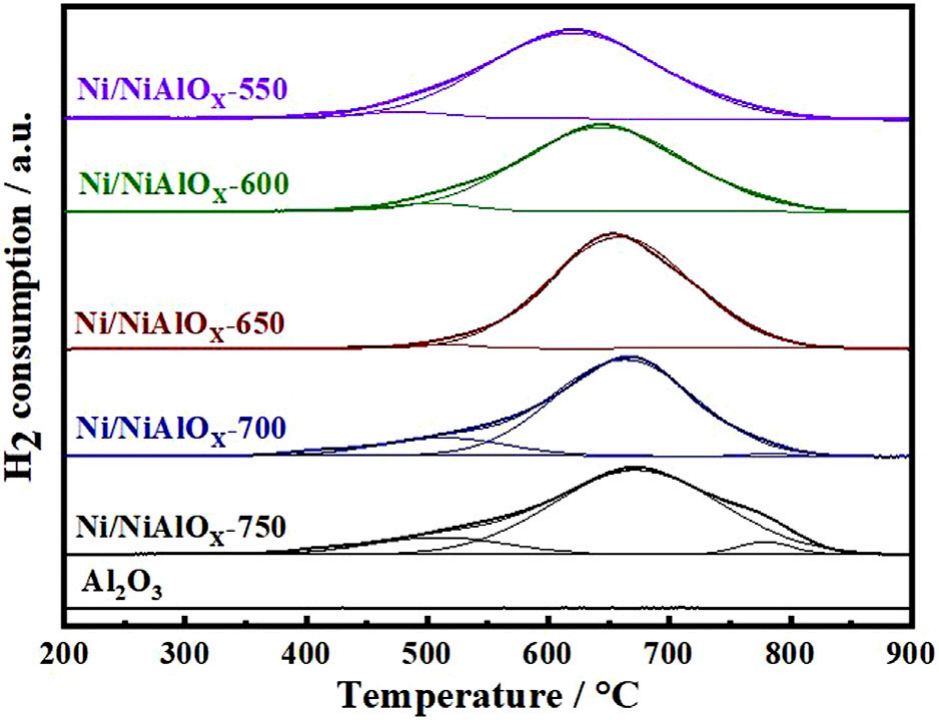
H_2_-TPR profiles of Ni/NiAlO_y_ catalysts with different calcination temperatures.

From the H_2_-TPR (Figure 2) analysis of calcined samples, the quantification of different Ni^2+^ was accomplished on the basis of Ni–support interaction (reducibility of different Ni^2+^ species). The H_2_-TPR data of bare Al_2_O_3_ prepared by the same modified sol–gel method was also given to make sure the observed TPR peaks belong to the reduction of Ni^2+^ species. In general, for the nickel aluminate samples, the reducible Ni^2+^ ions were categorized into four types by reduction temperatures, such as the bulk or free NiO, NiO bonded to Al_2_O_3_, and Ni^2+^ ions occupying the crystalline spinel structure of NiAl_2_O_4_ (Ni_octa_ or Ni_tetra_ sites) (Zhou et al., 2015; Li et al., 2019). According to the qualitative analysis of XRD and DR UV-Vis data discussed above, the different Ni^2+^ ions found in the prepared nickel aluminate samples could be free NiO or NiO bonded to nickel aluminate, Ni_octa_ and Ni_tetra_ of the NiAl_2_O_4_ spinel structure. As compared to the literature, the reduction temperature for NiO seemed higher in Figure 2, suggesting the interaction of formed NiO species with nickel aluminate (Yu et al., 2019). In comparison to samples calcined at other temperatures, the reduction peak corresponding to NiO was negligible for the sample calcined at 650^o^C. Furthermore, this particular sample calcined at 650^o^C contains more Ni_octa_ sites (98%) of the spinel structure according to the intensity of reduction peak observed at 658°C. For the samples calcined at the temperature above 650^o^C, the reduction peak corresponding to Ni_tetra_ appeared. Moreover, the increase in the intensity of reduction peak corresponding to NiO bonded to nickel aluminate was noticed. Thus, H_2_-TPR analysis proved the decrease of the inversion parameter in the spinel structure of nickel aluminate with the increase of calcination temperature above 650^o^C (O'Quinn et al., 2017).

#### Electronic Structure

**FIGURE 3:**
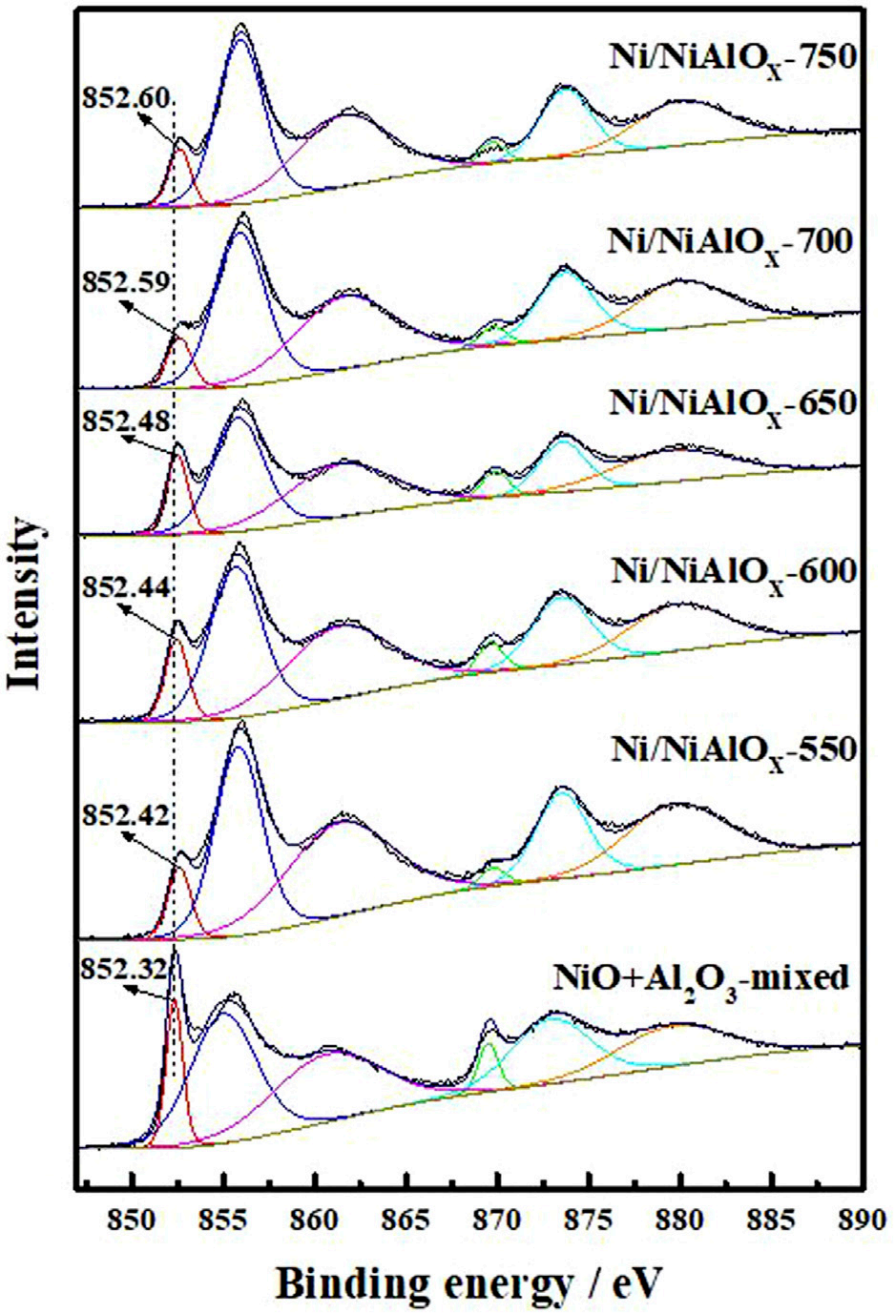
XPS spectra in the Ni $2P_{3/2}$ region of Ni/NiAlO_y_ catalysts with different calcination temperatures.

X-ray photoelectron spectroscopy analyses were carried out to identify the electronic state of Ni on the surface of reduced catalysts (Figure 3). The characteristic Ni^0^ 2p_3/2_, Ni^2+^ 2p_3/2_, and the satellite peak of Ni^2+^ 2p_3/2_ usually appeared at the binding energy of 852.5, 856.0, and 861.8 eV, respectively (Yancheshmeh et al., 2020). These three peaks were observed in the XPS analysis of the present catalysts. Compared to the sample prepared by mechanical mixing (852.32 eV), the distinctive peak of Ni^0^ 2p_3/2_ appeared at the higher binding energy for Ni/NiAlO_x_ catalysts due to their electron-deficient Ni sites. As was discussed above, the electron-deficient state of Ni sites in the prepared catalysts could be advantageous for promoting the activation of aromatics through adsorption.

### Catalytic Hydrogenation Performance

#### Hydrogenation Activity

**FIGURE 4:**
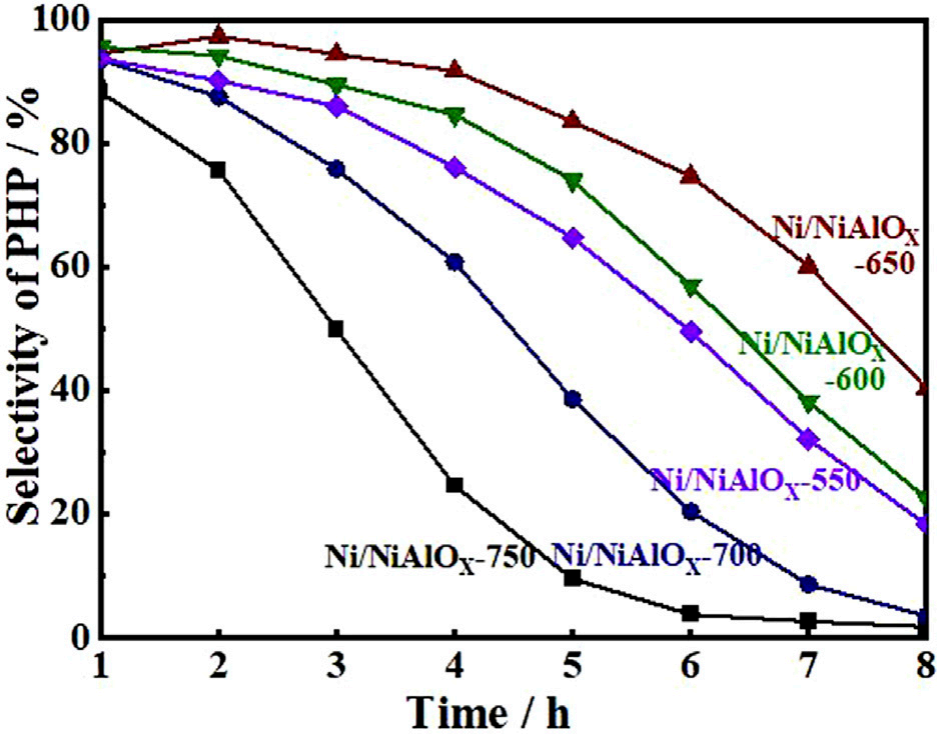
Selectivity of perhydro-phenanthrene over Ni/NiAlO_x_ catalysts calcinated at different temperatures.

Hydrogenation of PHE was performed under optimal reaction conditions (300°C, 5 MPa, a H_2_/oil volume ratio of 600, and WHSV of 52 h^−1^) to find out the best catalyst among the as-synthesized catalysts. The change in selectivity of PHP with reaction time during the hydrogenation of PHE over the attempted catalysts is shown in Figure 4, indicating PHP as the major product without the formation of any gas and ring opening products. All the Ni/NiAlO_X_ catalysts presented remarkable initial activity both in terms of PHE conversion (∼99%) and PHP selectivity (∼97%) even under high WHSV (52 h^−1^) due to the improved adsorption of aromatics that was promoted by the electron-deficient Ni sites. Ni/NiAlO_X_-650 reveals a comparatively higher hydrogenation activity than the other Ni/NiAlO_X_-T catalysts due to its metallic Ni sites predominantly produced from Ni_octa_ sites of the calcined form which expose the strong MSI. As evidenced by the CO-pulse adsorption data (Table 2), the strong MSI advantageously provided the higher Ni dispersion (1.99%) in Ni/NiAlO_x_-650 catalysts. The relatively higher S_meso_ of Ni/NiAlO_X_-650 (Table 1) could also contribute to the higher performance Ni/NiAlO_X_-650 in the hydrogenation of PHE.

**TABLE 2:** Metal dispersion and reactivity of Ni/NiAlO_X_ catalysts with different calcination temperatures.

Catalyst	Metal dispersion/%	Metallic surface area/m^2 ^g^−1^sample	Hydrogenation conversion of s-OHP^b^/ %	Observed reaction rate^b^×10^3^/ mol g^−1^s^−1^	TOF^c^×10^3^/ s^−1^
Ni/NiAlO_X_-550	1.73	4.66	6.55	0.32	2.69
Ni/NiAlO_X_-600	1.75	4.72	7.26	0.35	2.88
Ni/NiAlO_X_-650	1.99	5.36	13.24	0.64	4.65
Ni/NiAlO_X_-700	1.64	4.43	5.77	0.28	2.44
Ni/NiAlO_X_-750	1.32	3.55	3.96	0.19	2.08

a Calculated by CO pluse adsorption.

b Determined at 300°C, H_2_ pressure 5 MPa, 1 wt% s-OHP in decalin, WHSV 321 h^−1^.

c Turnover frequency (TOF), as described in *Catalytic Activity*.

#### Function of the Electron-Deficient State

**FIGURE 5:**
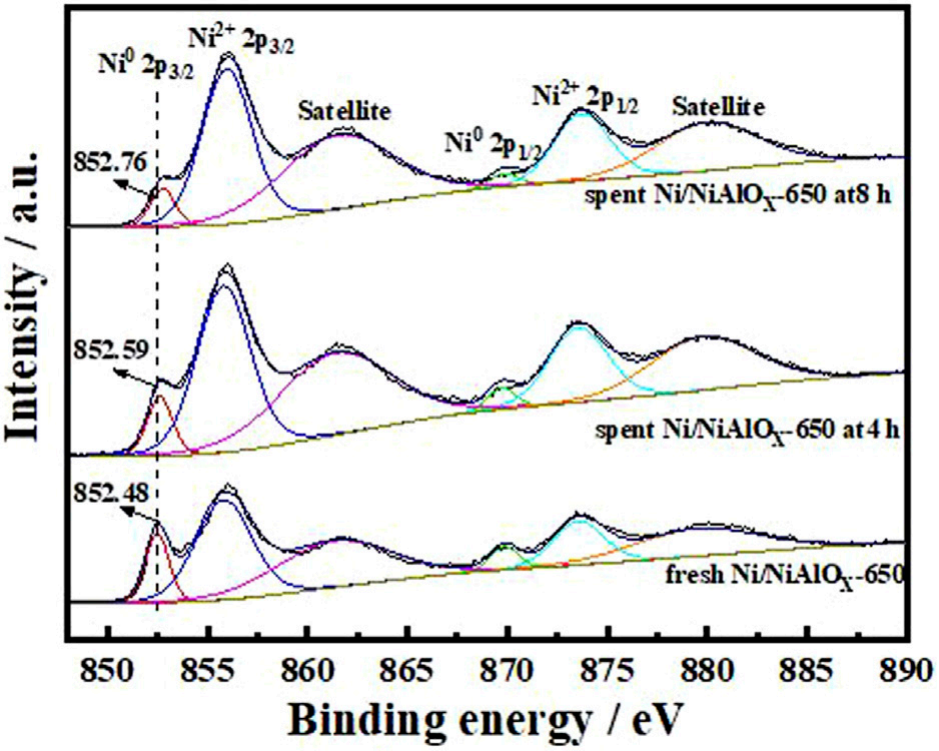
XPS spectra in the Ni $2P_{3/2}$ region of the Ni/NiAlO_x_-650 catalysts with different reaction times in the PHE hydrogenation process.

**FIGURE 6:**
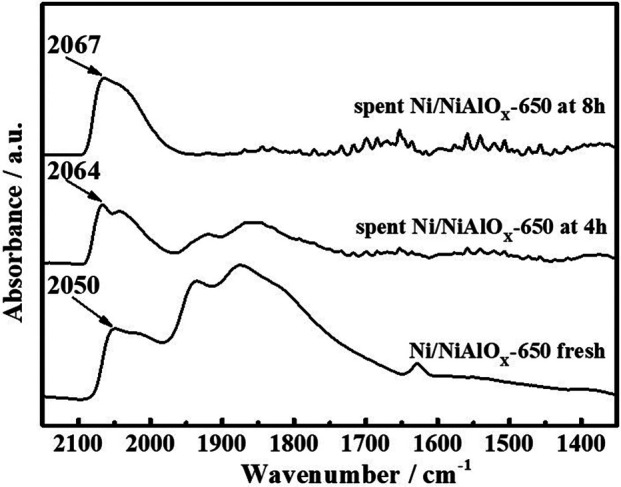
CO-FTIR of Ni/NiAlO_x_-650 catalysts with different reaction times in the PHE hydrogenation process.

To explain the gradual decrease of PHP selectivity (Figure 4) with an increase of s-OHP selectivity during the PHE hydrogenation, the characterization of spent catalyst was important. The comparative of XPS analysis of fresh and spent Ni/NiAlO_X_-650 was shown in Figure 5. The characteristic binding energy (Ni^0^ 2p_3/2_) corresponding to metallic Ni species has been shifted to higher binding energy for the spent Ni/NiAlO_X_-650 catalyst, which ranges from 852.4 to 852.7 eV, indicating the increase of electron deficiency of Ni^0^ sites during the PHE hydrogenation. For further insights about the role of the electronic structure of Ni^0^ species in PHE hydrogenation, the change in the electronic structure of surface Ni^0^ species after the PHE hydrogenation was studied by FT-IR analysis using CO as a probe molecule (Figure 6). In comparison, the upward shift, that is, 2050 cm^−1^ to 2064 cm^−1^ (spent catalyst at 4 h) and 2067 cm^−1^ (spent catalyst at 8 h), is observed in the characteristic stretch of linear CO absorption after PHE hydrogenation due to the weakening of interaction between surface Ni^0^ sites and CO (Yang et al., 2017). This can be attributed to the enhancing trend of electron deficiency of Ni^0^ sites during the PHE hydrogenation progress, which hampered the π-back bonding from Ni^0^ to CO (Lee and Oyama 2006), as supported by XPS results. Contrary to linear CO adsorption, no obvious shift was noticed in the peaks corresponding to the bridged CO adsorption (1936 cm^−1^ and 1874 cm^−1^) but the intensity of such peaks is apparently decreased. In particular, the bridged CO adsorption peak completely disappeared in the CO FT-IR spectrum of spent Ni/NiAlO_X_-650 catalysts for 8 h. This suggested that the Ni^0^ sites in the spent Ni/NiAlO_X_-650 catalysts became completely unsuitable to establish the CO absorption through bridging mode.

**FIGURE 7:**
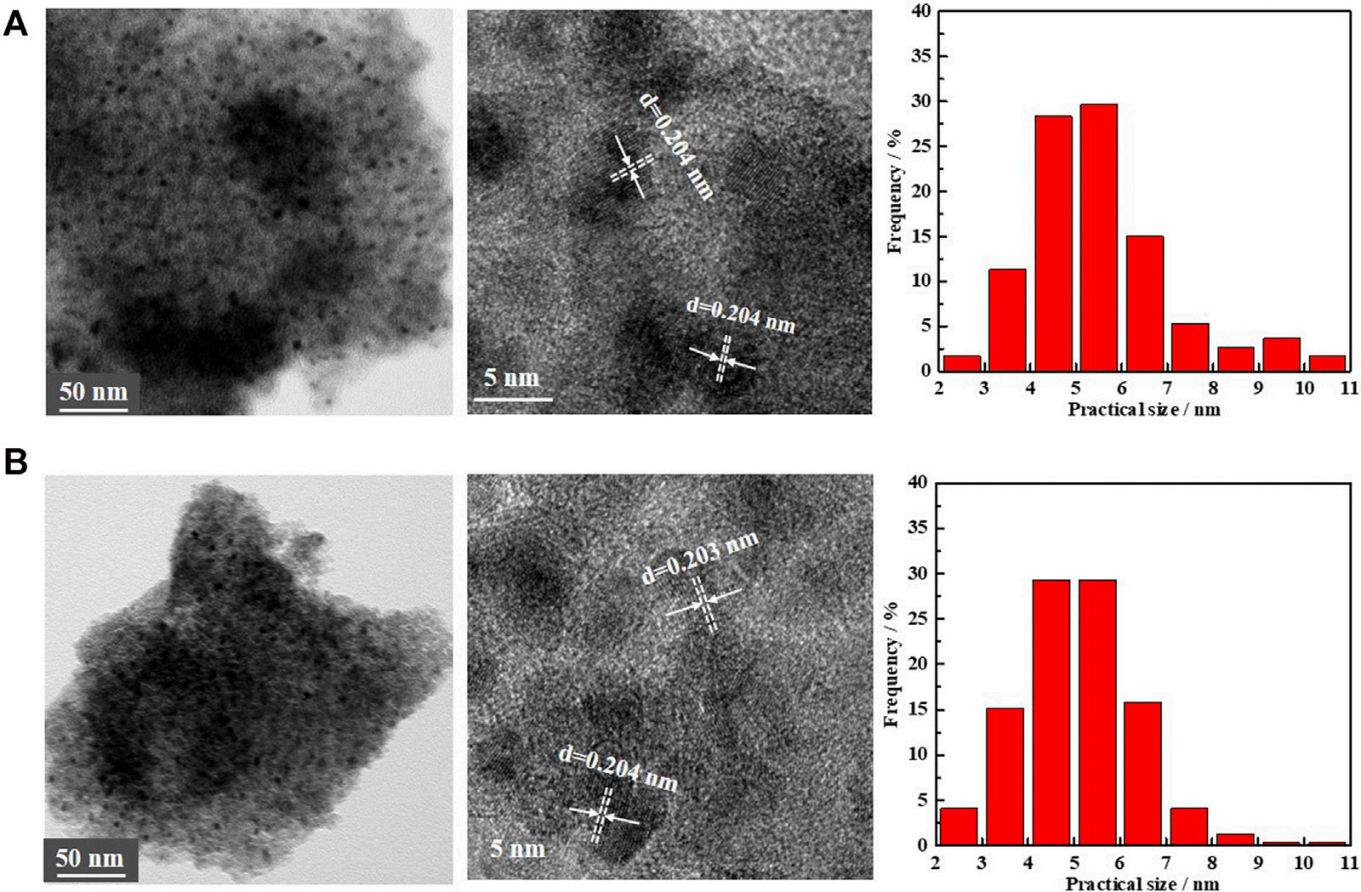
TEM images and particles size distribution of the **(A)** fresh Ni/NiAlO_x_-650 catalysts and **(B)** spent Ni/NiAlO_x_-650 catalyst.

To ensure that the decrease in activity during the progress of PHE hydrogenation occurred primarily by the change in the electronic structure, TEM analysis of the spent catalysts have been performed (Figure 7; Yang et al., 2017). According to TEM results, the fresh and spent Ni/NiAlO_X_-650 catalysts displayed the same pattern of lattice fringes with 0.204 nm lattice spacing that corresponds to Ni(111) plane, indicating that the geometric structure had no impact in inhibiting the PHE hydrogenation with time. Expecting the same adsorption mechanism for aromatics like CO over Ni^0^ sites (Lee and Oyama 2006), the charge transfer between surface Ni and aromatic rings could be reduced by the enhanced electron-deficiency of Ni^0^ sites, which was unfavorable for aromatic activation.

#### Hydrogenation Mechanism Exploration

**FIGURE 8:**
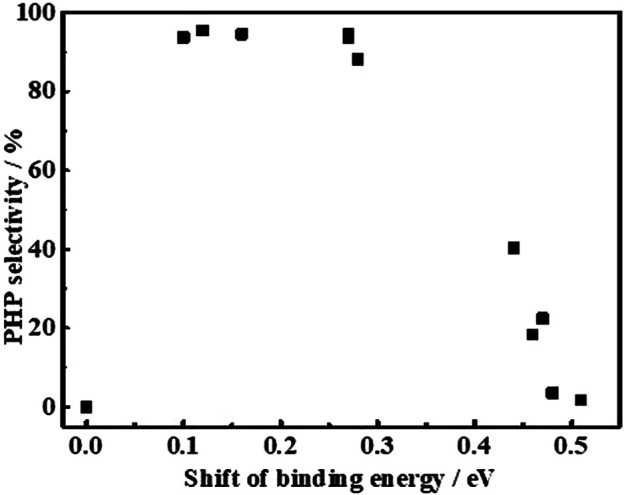
Relationship between shifts of Ni° $2P^{3/2}$ binding energy in XPS with the PHP selectivity.

To correlate the electronic properties of Ni^0^ sites with the PHP selectivity at different reaction times, a plot is made between the binding energy of Ni^0^ 2p_3/2_ in different catalysts (Figure 8). To support this study, a catalyst prepared from the reduction of a mechanical mixture containing NiO and Al_2_O_3_ (NiO + Al_2_O_3_-mixed) was taken as reference, whose binding energy (852.38 eV) was considered as a null point for the comparison to derive the shift in binding energy of Ni^0^ 2p_3/2_. As expected, the PHP selectivity was zero while using the NiO + Al_2_O_3_-mixed catalyst evaluated at the same hydrogenation conditions. For the comparison, the instant selectivity (1 h) as well as the end selectivity (8 h) of PHP while using different catalysts have been considered. According to Figure 8, the binding energy of Ni^0^ 2p_3/2_ in the range of 852.4–852.6 eV, that is, 0.1–0.3 eV shift from the actual binding energy of NiO + Al_2_O_3_-mixed, was essential, and that provided the favorable electron deficiency of Ni^0^ sites to enable better PHP selectivity (>90%). This big difference in activity caused by small changes in the electron state could be understood to seek the match of orbital energy levels. The electron-deficient state could improve the electronic donation from aromatics to Ni atom for σ bonding. However, an excessive deficient degree would limit the filling of electrons in antibonding orbitals to form π-back bonding.

The TEM image of spent Ni/NiAlO_X_-650 (Figure 7) indicated the nondisruptive dispersion of Ni^0^ active sites during the PHE hydrogenation, indicating the absence of sintering of Ni^0^ active sites. Meanwhile, the Raman spectrum of spent Ni/NiAlO_X_-650 catalysts for 8 h (Supplementary Figure S2) rules out the formation of carbon deposition on the catalyst surface during PHE hydrogenation. Therefore, it was confirmed that the decrease in PHE hydrogenation capacity of studied catalysts occurred primarily due to the unfavorable increase of electron deficiency in Ni^0^ active sites.

**FIGURE 9:**
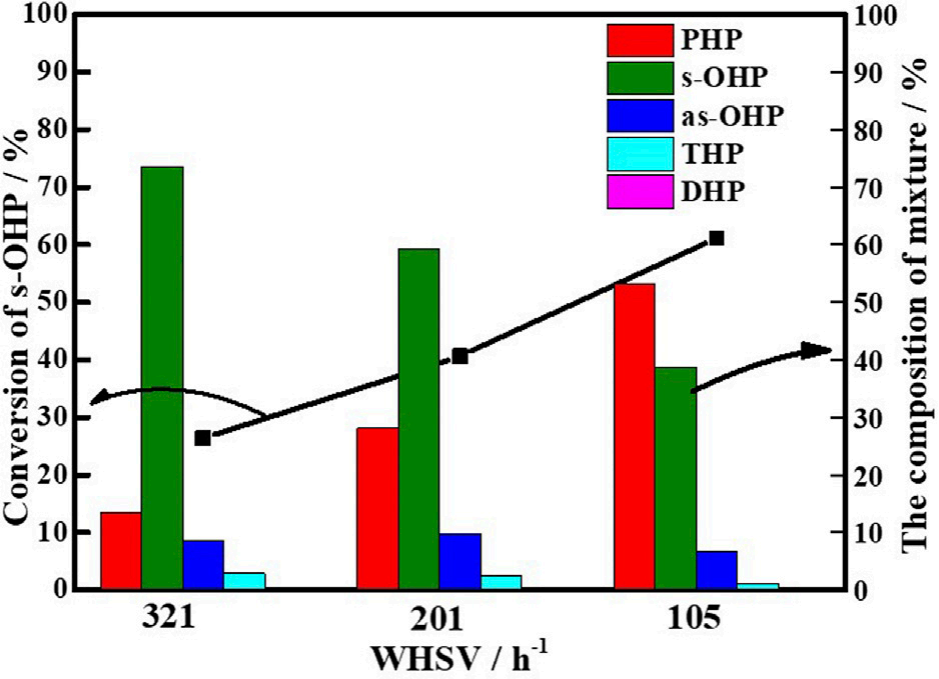
Hydrogenation of s-OHP over Ni/NiAlO_x_-650 catalysts with different weight hourly space velocity (WHSV).

The steric hindrance and competitive adsorption associated with the hydrogenation of s-OHP made it a major product in the main product of PHE hydrogenation Korre et al. (1995), Beltramone et al. (2008), Fu et al. (2015), Wang M et al. (2019), and thus, s-OHP conversion to PHP is regarded as the rate-determining step. Using s-OHP as the model compound, the real benefit of adequate electron deficiency in Ni^0^ sites in the hydrogenation reactions was highlighted. For the hydrogenation of s-OHP to PHP, the employed reaction conditions were as follows: 300°C, 5 MPa (H_2_), and H_2_/oil ratio of 600. The effects of internal and external diffusion were excluded by changing the mass flow rate and grain size, respectively. A carbon balance is attained over 96%. Figure 9 exhibited the change of s-OHP conversion and product selectivity with WHSV over Ni/NiAlO_X_-650 catalysts, representatively. Three simultaneous reaction processes of s-OHP over the Ni/NiAlO_X_-650 catalysts were predicted: saturation to PHP, isomerization to as-OHP, and dehydrogenation to THP and DHP. With the decrease of s-OHP, the content of dehydrogenation and isomerization yields in the product mixture was basically unchanged, but the content of PHP was gradually increased, which was almost equal to the conversion rate of s-OHP. These results proved that the conversion of s-OHP to PHP occurred through the hydrogenation route over Ni/NiAlO_X_-650 catalyst.

The obvious reaction rate (r_obs_) and the turnover frequency (TOF) in the performed s-OHP were calculated at the lower conversion (<20%) and given in Table 3. Ni/NiAlO_X_-650 showed a higher r_obs_ (0.64 × 10^−3^ mol kg^−1^ s^−1^) and TOF (4.65 × 10^−3^ s^−1^) than the other as-synthesized catalysts, and the activity trend of different catalysts was similar to that observed in the PHE hydrogenation. The higher efficiency of Ni/NiAlO_X_-650 could be assigned to the abundance of Ni_octa_ sites after its calcination, which promoted the dispersion of Ni^0^ sites and provided the more suitable electron deficiency.

The TOF of Ni/NiAlO_x_-650 in PHE hydrogenation was calculated and compared with that of the catalysts already reported in the literature (Table 3). Comparatively, Ni/NiAlO_X_-650 (WHSV of 52 h^−1^) accomplished a much higher average PHP selectivity (79.6%) than the other catalysts. For the r_obs_ and TOF calculation, WHSV was increased to 520 h^−1^ to obtain the lower PHE conversion (<20%). At this condition, the calculated value of r_obs_ and TOF is 1.53 × 10^−3^ mol kg^−1^ s^−1^ and 14.64 × 10^−^ s^−1^, respectively. These values were relatively higher than that reported for various catalysts, indicating the significance of Ni/NiAlO_X_-650 possessing the suitable electron-deficient Ni^0^ sites.

**TABLE 3:** Comparison of the catalytic performance of Ni/NiAlO_X_-650 with the literature in the deep hydrogenation of phenanthrene.

Reactor	Catalyst	Temperature/°C	H_2_ pressure/ MPa	WHSV/h^−1^	Conversion of PHE/ %	Selectivity/ %					r _obs_×10^3^/mol kg^−1^ s^−1^	TOF^a^×10^3^/s^−1^	Ref
						DHP	THP	as-OHP	s-OHP	PHP			
Fixed-bed	Ni/NiAlO_X_-650	300	5	52.0	98.9	0.7^b^	0.9^b^	2.8^b^	16.0^b^	79.6^b^	1.53	14.64	This work
	Ni(2.2)WS_2_	300	6	−	26.0	44.9	35.3	3.9	15.3	0	0.99	−	Luo et al. (2018)
	Ni(3)MoS_2_	300	6	−	−	−	−	−	−	−	0.43	−	Schachtl et al. (2015)
	NiMoS/MZSM-5	280	5	14.0	98.7	0	1.4	S_as-OHP_ + S_s-OHP_ = 80.7		17.9	0.74	1.80	Fu et al. (2015)
	MoP/ZSM-5-NA	290	5	15.4	77.0	S_as-OHP_ + S_s-OHP_ + S_PHP_ = 43.0					0.34	0.07	Fu et al. (2019)
	Ni_2_P/HZSM-5-M	300	5	15.4	99.0	S_as-OHP_ + S_s-OHP_ + S_PHP_ = 98.0					1.32	8.20	Fu et al. (2016)
	Pt (3)-Pd (10)	320	5	28.0	97.2	2.0	4.0	2.0	10.0	82.0	1.20	−	Qian et al. (1999)
Autoclave	NiO(55%)/Al_2_O_3_	315	7.8		97.9	16.9	7.4	15.4	39.4	20.8	−	−	Nuzzi and Marcandalli (2003)
	Ni-PSNT	240	4		100	0	9.7	6.0	58.8	25.5	−	−	Wang D et al. (2019)

a Detected at total phenanthrene conversion lower than 20%.

b Average selectivity in phenanthrene hydrogenation for 8 h.

## Conclusion

The electronic properties of relatively low-cost metallic active site (Ni) were tuned by the facile calcination method to attain the improved activity with a better selectivity of perhydro-products during phenanthrene hydrogenation. It was found that nickel occupation in the nickel aluminate–based catalyst could be regulated by varying the calcination temperatures (550-750^o^C) of the catalyst’s precursor. When the calcination temperature was 650°C, around 98% octahedral Ni^2+^ sites were formed, which were relatively easily reduced and constructed the favorable metal–support interaction that improved the electron deficiency degree of Ni active sites in the catalyst (Ni/NiAlO_x_-650) obtained after reduction. Phenanthrene hydrogenation evaluation showed that Ni/NiAlO_x_-650 displayed the improved initial selectivity of perhydrophenanthrene (98%) along with a higher TOF (1.53 × 10^−3^ mol kg^−1^ s^−1^) at 300°C, 5 MPa (H_2_). The characterization of the spent catalysts indicated that electron-deficient degree of Ni active sites played an important role in phenanthrene hydrogenation, which overcomes the steric hindrance and competitive adsorption of octahydrophenanthrene.

## Data Availability

The original contributions presented in the study are included in the article/Supplementary Material; further inquiries can be directed to the corresponding authors.
