# Supplementary material for: Synthesis of Ni/NiAlOx Catalysts for Hydrogenation Saturation of Phenanthrene
Source: Front Chem. 2021 Oct 8;9:757908. doi: 10.3389/fchem.2021.757908 (PMC8531806; doi:10.3389/fchem.2021.757908)
Supplement: Supplementary file 1 [file DataSheet1.pdf]

Synthesis of Nickel Aluminate-based Catalysts for Hydrogenation Saturation of Phenanthrene

Dao-Cheng Liu[1, 2], Yu Chen[1, 2], Jie-Ying Jing[1, 2,*], Antony Rajendran[1, 2], Hong-Cun Bai[3], Wen-Ying Li[1, 2, *]

[1] State Key Laboratory of Clean and Efficient Coal Utilization, Taiyuan University of Technology, Taiyuan 030024, China

[2] Key Laboratory of Coal Science and Technology (Taiyuan University of Technology), Ministry of Education, Taiyuan 030024, China

[3] State Key Laboratory of High-efficiency Utilization of Coal and Green Chemical Engineering, Ningxia University, Ningxia 750021, PR China

* Corresponding authors. Tel. & fax: 86-351-6018453

Email address: jingjieying@tyut.edu.cn (for Jie-Ying Jing); ying@tyut.edu.cn (for Wen-Ying Li)

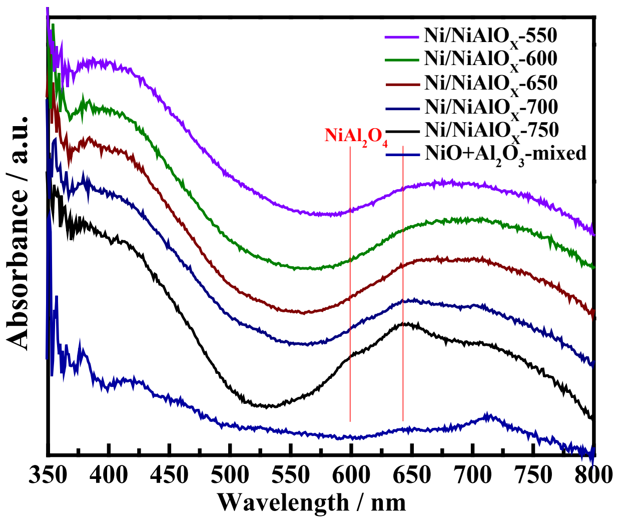

Figure S1. UV–VIS DRS spectra of Ni/NiAlO$_X$ catalysts after calcination at different calcination temperatures.

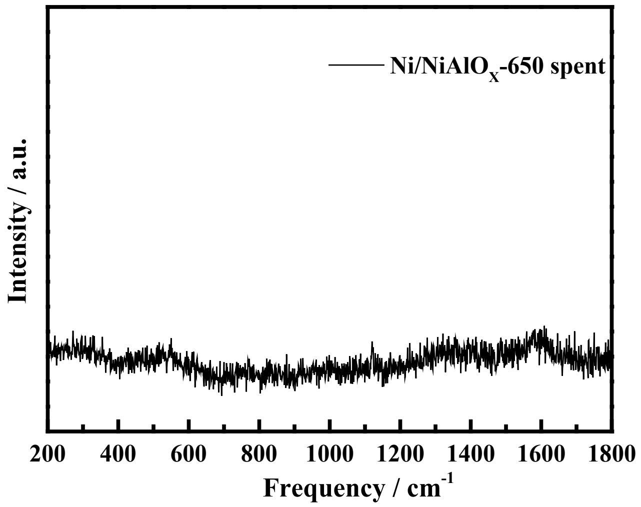

Figure S2. Raman spectroscopy curve of spent Ni/$NiAlO_x$-650 catalyst
